# Complex structural rearrangements are present in high-grade dysplastic Barrett’s oesophagus samples

**DOI:** 10.1186/s12920-019-0476-9

**Published:** 2019-02-04

**Authors:** Felicity Newell, Kalpana Patel, Michael Gartside, Lutz Krause, Sandra Brosda, Lauren G. Aoude, Kelly A. Loffler, Vanessa F. Bonazzi, Ann-Marie Patch, Stephen H. Kazakoff, Oliver Holmes, Qinying Xu, Scott Wood, Conrad Leonard, Guy Lampe, Reginald V. Lord, David C. Whiteman, John V. Pearson, Katia Nones, Nicola Waddell, Andrew P. Barbour

**Affiliations:** 1 QIMR Berghofer Medical Research Institute, 300 Herston Road, Herston, Brisbane, QLD 4006 Australia; 2 Surgical Oncology Group, Diamantina Institute, The University of Queensland, Translational Research Institute, Woolloongabba, Brisbane, QLD 4102 Australia; 3 The University of Queensland, Diamantina Institute, Translational Research Institute, Woolloongabba, Brisbane, QLD 4102 Australia; 4 College of Medicine and Public Health, Flinders University, Bedford Park, Adelaide, SA 5042 Australia; 5 Department of Anatomical Pathology, Princess Alexandra Hospital, Woolloongabba, Brisbane, Queensland 4102 Australia; 6 Department of Surgery, School of Medicine, University of Notre Dame, Sydney, Australia; 7 St. Vincent’s Centre for Applied Medical Research and University of New South Wales, Suite 606, 438 Victoria Street, Darlinghurst, Sydney, NSW 2010 Australia; 8 Upper Gastro-intestinal Surgical Unit, Department of Surgery, Princess Alexandra Hospital, Woolloongabba, Brisbane, QLD 4102 Australia

**Keywords:** Oesophageal adenocarcinoma, Barrett’s oesophagus, Chromothripsis, Breakage-fusion bridge, Whole-genome sequencing

## Abstract

**Background:**

Oesophageal adenocarcinoma (EAC) incidence is increasing and has a poor survival rate. Barrett’s oesophagus (BE) is a precursor condition that is associated with EAC and often occurs in conjunction with chronic gastro-oesophageal reflux, however many individuals diagnosed with BE never progress to cancer. An understanding of the genomic features of BE and EAC may help with the early identification of at-risk individuals.

**Methods:**

In this study, we assessed the genomic features of 16 BE samples using whole-genome sequencing. These included non-dysplastic samples collected at two time-points from two BE patients who had not progressed to EAC over several years. Seven other non-dysplastic samples and five dysplastic BE samples with high-grade dysplasia were also examined. We compared the genome profiles of these 16 BE samples with 22 EAC samples.

**Results:**

We observed that samples from the two non-progressor individuals had low numbers of somatic single nucleotide variants, indels and structural variation events compared to dysplastic and the remaining non-dysplastic BE. EAC had the highest level of somatic genomic variations. Mutational signature 17, which is common in EAC, was also present in non-dysplastic and dysplastic BE, but was not present in the non-progressors. Many dysplastic samples had mutations in genes previously reported in EAC, whereas only mutations in *CDKN2A* or in the fragile site genes appeared common in non-dysplastic samples. Rearrangement signatures were used to identify a signature associated with localised complex events such as chromothripsis and breakage fusion-bridge that are characteristic of EACs. Two dysplastic BE samples had a high contribution of this signature and contained evidence of localised rearrangements. Two other dysplastic samples also had regions of localised structural rearrangements. There was no evidence for complex events in non-dysplastic samples.

**Conclusions:**

The presence of complex localised rearrangements in dysplastic samples indicates a need for further investigations into the role such events play in the progression from BE to EAC.

**Electronic supplementary material:**

The online version of this article (10.1186/s12920-019-0476-9) contains supplementary material, which is available to authorized users.

## Background

The incidence of oesophageal adenocarcinoma (EAC) is increasing in Western countries and the long-term survival rate for the cancer is poor, with a 5-year survival rate of 14% [1, 2]. The strongest risk factor for developing EAC is being diagnosed with the precursor condition, Barrett’s oesophagus (BE) [3]. BE involves the replacement of the stratified squamous epithelium that is usually present in the distal oesophagus with metaplastic columnar epithelium and is associated with chronic gastric reflux [4]. BE has been proposed to involve progression through a number of histologic stages starting with a non-dysplastic metaplastic stage, through low-grade dysplasia (LGD), high-grade dysplasia (HGD) before leading to the development of EAC [5]. However, the majority of patients diagnosed with BE do not go on to develop EAC and are termed non-progressors [6]. It is estimated that 0.12–0.33% of patients diagnosed with BE will progress to EAC annually [7, 8]. The molecular basis for the progression of BE to EAC in those individuals who develop cancer, termed progressors, is not yet fully understood.

In recent years, a number of studies have used next-generation sequencing data to characterise the molecular characteristics of EAC and the changes that are associated with progression of BE to EAC. EAC has been shown to have a high mutation burden of single nucleotide variants (SNVs) of 8–10 mutations per megabase [9–11], making it one of the most highly mutated cancers after lung cancer and melanoma [12]. The majority of EACs have mutations in the tumour suppressor *TP53*, and other recurrently mutated genes include *SMAD4* and *ARID1A* [9]. Although mutations in tumour suppressor genes are common, recurrent SNVs or indels in oncogenes are rare, indicating that other mechanisms must be at work to activate oncogenes to drive cancer progression. BE has also been found to have a high mutation burden, with numbers higher than some invasive cancers, including breast and pancreatic cancer [13]. Mutations in genes such as *ARID1A*, *SMARCA4* and *TP53* have been identified in non-dysplastic and dysplastic BE [10, 13, 14].

When mutational signature analysis is performed using EAC samples, the presence of signature 17 is common. This signature is characterised by T:A > G:C transversions within a CTT tri-nucleotide context and has been proposed to arise from oxidative damage due to gastrointestinal reflux, although this has not yet been definitively proven [9, 11]. Signature 17 has also been reported to be present at similar levels in BE samples to that observed in EAC [10, 13]. Other mutational signatures are also present in EAC. Recently it has been proposed that the presence of mutation signature 3 is important for disease progression in a subset of samples. This signature is associated with *BRCA1* and *BRCA2* mutations and is indicative of defective homologous recombination [15]. Little is known about the presence of mutational signatures such as signature 3 in BE samples.

Genomic instability is common in EAC, with many EAC samples having high numbers of structural variation events and a significant proportion of the genome affected by copy number alterations [11]. Whole-genome doubling events and telomere shortening occurs frequently [13, 16]. In a recent whole-genome sequencing study of 129 EACs, it was observed that recurrently mutated genes were more often affected by rearrangements, amplifications and deletions than SNVs or indels [15]. Genes that were recurrently affected by rearrangements included *CDKN2A*, *SMYD3, RUNX1, CTNNA3, ERBB2, EGFR, MDM2* as well as fragile site genes *WWOX* and *FHIT*. In comparison with EAC, BE samples have a lower percentage of the genome affected by copy number changes, and loss of *CDKN2A* has been identified as an early event in BE [10, 13]. In a longitudinal case-cohort study, Li and co-workers observed that individuals with BE who did not progress to EAC (non-progressors) had relatively stable genomes over time, whereas BE patients who progressed to EAC developed chromosomal instability with initial copy number gains and losses followed by whole-genome doubling [17]. Telomere shortening has also been observed across the different histological grades of BE [18].

Catastrophic genomic events such as chromothripsis and breakage-fusion-bridge (BFB) are common in EAC. We have previously demonstrated that almost a third of EAC cases examined had such a genomic catastrophe and that these events often lead to the amplification of oncogenes such as *MDM2, MYC* and *KRAS* [11]. The presence of localised regions of hypermutation, termed kataegis, were also identified. Secrier et al. also observed that such events were common, with many samples showing evidence of complex rearrangements (32%), chromothripsis (30%) or kataegis (31%) [15]. Little is known about the prevalence of catastrophic events in BE. Using SNP arrays, Li and co-workers identified that 16% of BE patients who later progressed to EAC had evidence of chromothripsis prior to the detection of cancer [19]. However, no information about kataegis or the incidence of other complex events such as BFB are known. To date, no whole-genome sequenced BE samples have been examined to determine whether genomic catastrophes are present in these pre-cancer lesions.

A number of models have been proposed for the molecular progression of Barrett’s oesophagus to EAC. A linear model of progression has been proposed to involve the early loss of *CDKN2A*, followed by mutations in *TP53* in turn leading to increases in copy number and genome doubling [17, 20–24]. Stachler et al. proposed two potential pathways for development, with one involving gradual accumulation of losses in tumour suppressor genes, followed by activation of oncogenes and genomic instability [13]. The second pathway is proposed to involve loss of *TP53* as an early event followed by genome doubling and genomic instability, oncogene activation and aneuploidy [13]. The role of genomic catastrophes in models of progression is poorly understood. We have previously postulated that genomic catastrophes that lead to amplification of oncogenes could be one way that BE may rapidly undergo progression to EAC [11].

The aim of this study was to characterise the genomic landscape of 16 BE from 14 donors, including 2 non-dysplastic samples from 2 non-progressors, 7 other non-dysplastic and 5 dysplastic samples and compare them with the genomic features of 22 EAC samples. In particular, the presence of localised complex structural rearrangements in BE samples was determined.

## Methods

### Samples and DNA extraction

Samples used in this study were obtained from patients who have given their written, informed consent and with approval from the Metro South Health research ethics committee. Samples were obtained from 14 patients with Barrett’s oesophagus, with a total of 16 samples collected. BE samples were obtained from patients undergoing endoscopy at the Princess Alexandra Hospital, Brisbane, Australia. The biopsy sample site was recorded prospectively. Haematoxylin and eosin (H&E) slides were examined by a qualified pathologist (GL) to determine the stage of the BE samples. Biopsies collected at the same location as those used for H&E were snap frozen and stored prior to extraction of DNA. Biopsies used for DNA extraction in the study were sectioned prior to thawing and one piece used for histological to confirm the presence of goblet cells as an indication of epithelial tissue in the same sample. Representative images of H&E slides for Barrett’s samples are presented in Additional file 1: Figure S8. Matched normal samples were also obtained from each patient and were either blood or adjacent endoscopically and histologically normal tissue. DNA was extracted and quantified using Qubit (Thermo Fisher Scientific, Waltham, Massachusetts, MA, USA). Twenty-two oesophageal adenocarcinoma (EAC) samples were analysed for comparison. The collection and clinical information for these samples have previously been described [11]. Clinical and sample collection information for the cohort is available in Table 1 and Additional file 2: Table S1a (EAC samples) and 1b (BE samples).

**Table 1:** Clinical characteristics of cohort

ID	Age	Sex	Sample type
NP-1-1	77	M	NON-PROGRESSOR
NP-1-2	80	M	NON-PROGRESSOR
NP-2-1	58	M	NON-PROGRESSOR
NP-2-2	62	M	NON-PROGRESSOR
NDBE-1	64	M	NON-DYSPLASTIC
NDBE-2	67	M	NON-DYSPLASTIC
NDBE-3	66	M	NON-DYSPLASTIC
NDBE-4	56	M	NON-DYSPLASTIC
NDBE-5	79	M	NON-DYSPLASTIC
NDBE-6	60	M	NON-DYSPLASTIC
NDBE-7	78	M	NON-DYSPLASTIC
HGD-1	39	M	DYSPLASTIC (HIGH GRADE)
HGD-2	71	M	DYSPLASTIC (HIGH GRADE)
HGD-3	67	M	DYSPLASTIC (HIGH GRADE)
HGD-4	81	M	DYSPLASTIC (HIGH GRADE)
HGD-5	67	M	DYSPLASTIC (HIGH GRADE)
EAC-1	46	M	EAC
EAC-2	58	M	EAC
EAC-3	74	M	EAC
EAC-4	64	M	EAC
EAC-5	59	F	EAC
EAC-6	71	M	EAC
EAC-7	77	M	EAC
EAC-8	27	M	EAC
EAC-9	54	M	EAC
EAC-10	48	M	EAC
EAC-11	59	M	EAC
EAC-12	72	M	EAC
EAC-13	65	M	EAC
EAC-14	57	M	EAC
EAC-15	64	M	EAC
EAC-16	74	M	EAC
EAC-17	70	M	EAC
EAC-18	77	M	EAC
EAC-19	49	M	EAC
EAC-20	68	M	EAC
EAC-21	58	M	EAC
EAC-22	75	M	EAC

### Whole-genome sequencing

EAC samples were sequenced previously [11]. BE samples underwent whole-genome paired-end sequencing on a HiSeq2000 or a X-Ten (Illumina, San Diego, CA, USA) at one of two facilities: The Kinghorn Cancer Centre, Garvan Institute of Medical Research (Sydney, Australia) or Macrogen (Geumcheon-gu, Seoul, South Korea). EAC samples underwent reanalysis (ie. sequence alignment and variant calling) in order to allow direct comparison between BE and EAC samples. All samples were aligned to the human genome assembly GRCh37 using BWA-MEM. Mean coverage was determined using qCoverage (available at http://sourceforge.net/projects/adamajava). EAC samples had a mean coverage of 76 (range 63–135), BE samples had a mean coverage of 81 (range 61–134) and matched normal samples had a mean coverage of 38 (range 30–69) (Additional file 2: Table S1c). All samples had a minimum tumour or Barrett’s epithelium percentage of 20%, as determined by ascatNGS (Additional file 2: Table S1c).

### SNV and indel calling

SNV calling was carried out using a dual calling strategy using the consensus of two different tools: qSNP [25] and GATK [26] as previous described [11]. Indel calling (1-50 bp) was carried out using GATK. Variant annotation for gene consequence was performed using SnpEff and the Ensembl gene annotation [27]. To compare overlapping variants in the non-progressor samples NP-1 and NP-2, pileups were performed at each variant position identified in the variant calling pileup using qbampileup (available at http://sourceforge.net/projects/adamajava) in order to look for weak evidence for variants in each sample. A variant was considered to be present when there were no alternate alleles present in the normal sample, when there were at least 10 reads of coverage and if there was at least one good quality read in the BE sample. Reads that were not considered to be high quality were: duplicate reads, reads with mapping quality < 10, reads with CIGAR score < 34 or greater than 3 mismatches in the read.

### Mutational signatures

Mutational signatures were detected using the non-negative matrix factorization (NMF) method described by Alexandrov et al. [12]. EAC and BE samples were analysed together. Signatures obtained using NMF were compared with the known signatures described in the COSMIC database using cosine similarity (http://cancer.sanger.ac.uk/cosmic/signatures, accessed 27 October 2017). To determine the contribution of each signature to a sample, we used the quadratic programming approach available in the R package, SignatureEstimation [28]. To prevent over-fitting, signatures that contributed less than 10% for a sample were removed and mutations were reassigned to the signatures that remained.

### Kataegis

Localised regions of hypermutation, known as kataegis, were identified as previously described [11]. Briefly, inter-mutational distance was calculated as the number of base pairs between mutations ordered by chromosome and position. Inter-mutational distances were segmented using piecewise constant fitting and putative regions of kataegis were defined as segments containing six or more consecutive mutations with a mean inter-mutation distance of ≤1000 bp.

### Copy number and structural variant analysis

Copy number was determined using sequencing data and the tool ascatNGS [29]. In order to be conservative in the analysis we only considered high level amplifications, homozygous deletions or regions with significant gain or loss in a duplicated genome, in a similar manner to that described in the COSMIC database (https://cancer.sanger.ac.uk/cosmic/help/cnv/overview). The following definitions were using for copy number amplification or deletion. An amplification was called with a region having either: i) average genome ploidy <= 2.7 AND total copy number > = 6 OR ii) average genome ploidy > 2.7 AND total copy number > = 9. A region of loss was called if either i) average genome ploidy <= 2.7 AND total copy number = 0 OR ii) average genome ploidy > 2.7 AND total copy number < (average genome ploidy - 2.7). Copy number per gene was determined by annotation against Ensembl known genes (version 75). Structural variants were determined using qSV as previously described [11].

### Rearrangement signatures

We used the same statistical framework using NMF that was used for mutational signature analysis for the identification of rearrangement signatures [12]. SVs were classified into the same categories as has been previously described and applied to a breast cancer cohort by Nik-Zainal and co-workers [30]. SVs were classified into types of events: deletions, duplications, inversions and inter-chromosomal translocations. SVs were further characterised by size and whether the breakpoints were clustered or non-clustered. Size categories (for events that were not translocations) were: 1–10 kb, 10–100 kb, 100 kb–1 Mb, 1–10 Mb, more than 10 Mb. Clustered SV breakpoints were defined using the BEDTools cluster function [31]. Clustered events were defined using the presence of ≥10 breakpoints in a 1 Mb window, a metric that has previously been applied by Letouze et al. [32].

### Identification of localised complex events

For EAC and BE samples, samples were identified as having patterns similar to chromothripsis or breakage-fusion-bridge (BFB) by manual review of each chromosome using a combined plot of copy number LogR ratio (LRR) and B allele frequency (BAF), copy number events and structural events. Evidence for complex events was determined by looking for: 1) the features of chromothripsis as described by Korbel and Campbell [33] including oscillating copy number, random joins, retention of heterozygosity; 2) evidence of BFB including telomeric loss and a local region of amplification with a high number of inversions; and 3) localised region of complexity including regions with clustered SV breakpoints that also had several changes in copy number and/or retention of heterozygosity or a region that was highly amplified (copy number > 9).

### Telomere length

Telomere length was determined using sequencing data and the tool qMotif, which is available at http://sourceforge.net/projects/adamajava and as previously described [34]. qMotif counts the number of reads containing the telomeric repeat (TTAAGG). Counts are normalised to the mean genomic coverage of a sample and the relative telomere length is expressed as the log2 ratio of read counts in the EAC/BE sample BAM file to the matched normal BAM file read counts.

## Results

### Comparison of genomic features of BE and EAC

We have previously reported the genomic features of 22 EAC samples that underwent whole-genome sequencing [11]. Here, we compared the somatic events of these EAC samples with 16 BE samples from 14 patients with different histological stages on a genome-wide scale. Clinical information for the cohort are detailed in Table 1 and Additional file 2: Tables S1a (EAC samples) and S1b (Barrett’s samples). The BE samples were comprised of samples from 2 non-progressor patients (NP-1 and NP-2) with BE that had not progressed to cancer over a number of years. For each non-progressor we examined 2 samples which were taken 3 years apart for patient NP-1 and 4 years apart for patient NP-2 (Additional file 1: Figure S1). We also characterised 7 non-dysplastic BE and 5 dysplastic BE samples. All dysplastic samples were determined histologically to be HGD. Five of the non-dysplastic samples, and one of the dysplastic samples, were from patients with EAC and biopsies were taken from a region adjacent to, but well separated from, the tumour at the time of surgery. All BE and EAC samples underwent paired-end whole-genome sequencing to a mean base pair depth of 81x (BE) and 76x (EAC), and matched normal samples (blood or adjacent endoscopically normal oesophageal/gastric sample) to a mean base pair depth of 38x. The coverage for each sample is listed in Additional file 2: Table S1c.

**Fig. 1:**
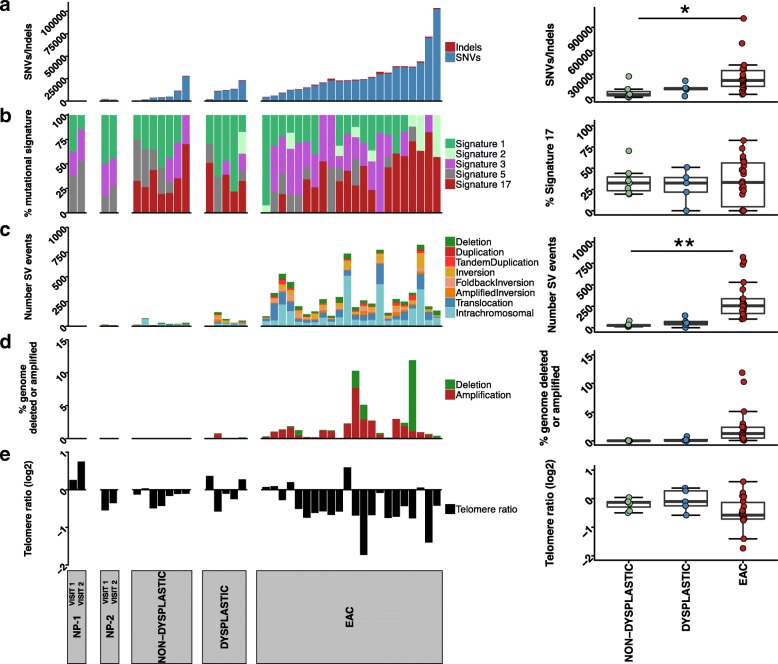
Somatic variants, mutational signatures and telomere length in BE and EAC. Barcharts and boxplots of: (**a)** number of SNP/indels in, from left to right, non-progressors (NP-1 and NP-2), non-dysplastic BE, dysplastic BE, and EAC samples; (**b)** Proportion of each mutational signature: Signature 1,2,3,5,17 (left) and boxplot of proportion of Signature 17 in non-dysplastic, dysplastic and EAC samples; (**c)** Number of SV events; (**d**) Percent of the genome affected by copy number deletion or amplification (**e**) Telomere length. In the boxplots, ANOVA was used to determine significance (*P < 0.05, **P < 0.001). Sample order for the figure can be found in Additional file 2: Table S1c

As has been previously described [11], the EAC samples exhibited high numbers of somatic SNVs/indels, with a median of 7.33 mutations/Mb (range = 1.40–33.85) (Fig. 1 a). When comparing EAC, dysplastic and non-dysplastic BE samples, a significant difference in somatic mutations was observed (ANOVA, *P* = 0.033). Dysplastic BE samples had a median of 3.9 mutations/Mb (range = 0.66–7.23), and non-dysplastic samples had a median of 1.28 mutations/Mb (range = 0.12–9.10). There was a significant difference observed between EAC and non-dysplastic samples (post hoc Tukey HSD *P* = 0.048), but no significant difference between the mutation burden of dysplastic BE and EAC (post hoc Tukey HSD *P* = 0.21) or between non-dysplastic and dysplastic BE (post hoc Tukey HSD *P* = 0.92). If only coding mutations were examined a similar pattern was observed with an overall significant difference (ANOVA, *P* = 0.002), but no significant difference between EAC and dysplastic samples (post hoc Tukey HSD *P* = 0.12) or non-dysplastic and dysplastic samples (post hoc Tukey HSD *P* = 0.52).

To determine if non-progressor samples were genomically stable over time, we compared the SNVs present within BE samples collected at two time points for the donors NP-1 and NP-2. (Additional file 1: Figure S2). Pileups were performed at each position in order to consider weak evidence for each mutation where there was strong evidence in one of the samples. Using this approach there was good overlap for both donors, with 79% of mutations for NP-1 and 90% of mutations for NP-2 with evidence in both samples.

Mutational signature analysis was performed by analysing the BE and EAC samples together using the framework described by Alexandrov (Fig. 1 b) [12]. Five mutational signatures were observed, and were compared to the signatures reported in the COSMIC database using the cosine similarity (Additional file 1: Figure S3a,b). The signatures identified were: Signature 17; Signature 3, which is associated with BRCA1 and BRCA2 mutations and homologous recombination defects; Signature 1, which is associated with aging; Signature 5, of unknown aetiology; and Signature 2, which is attributed to APOBEC activity. The signatures within the EAC samples were consistent with those previously reported using these EAC samples, with the previously novel signature now assigned to Signature 5 by cosine similarity [11].

Signature 17 is common in EAC samples (Fig. 1 b). This signature is present in 21 of the 22 EAC samples, with a mean of 34% of the mutations in EAC samples attributed to this signature (range: 0–82.6%). It is also present at similar levels in the dysplastic and non-dysplastic BE samples, with a mean of 28.9 and 35.5% respectively, with no significant difference between the groups (ANOVA, *P* = 0.83). There was no statistically significant difference between the contributions of Signature 5 (*P* = 0.35), 2 (*P* = 0.22), 3 (*P* = 0.05) or 1 (*P* = 0.06). However, the overall trend was for lower levels of Signature 1 in EAC samples (mean of 21.9%) compared with non-dysplastic and dysplastic BE samples (mean of 31.4 and 46.9% respectively), and higher levels of Signature 3 in EAC samples (mean of 28%) compared with BE samples (non-dysplastic mean of 13% and dysplastic mean of 6.2%). Non-progressor samples showed no evidence of Signature 17, with mutations in these samples assigned to Signatures 1, 5 and 3. The proportion of each signature was similar when comparing the Visit 1 and Visit 2 samples for both non-progressors (NP-1 and NP-2).

Structural variant events (SV) were identified using qSV and copy number variants (CNV) identified using ascatNGS. EAC samples had high numbers of SV events, with a mean number per sample of 315 (range 101–824) (Fig. 1 c) and a mean of 28% of the genome affected by copy number deletions (copy number 0 or 1) or amplifications (copy number > =6) (Fig. 1 d). Dysplastic BE samples had a mean of 62 SV events per sample (range of 2–142) and between 1 and 14.5% of the genomes affected by copy number changes. Non-dysplastic BE samples had a mean of 33 SV events (range of 12–81) and few copy number changes, with a mean of 0.7% of the genome affected. The overall occurrence of SV events was statistically different between the three groups (ANOVA, *P* = 0.0009), but no difference was observed between non-dysplastic and dysplastic BE samples (post hoc Tukey HSD *P* = 0.95). The overall difference in percentage of the genome affected by copy number deletions or amplification was not statistically different between the three groups (ANOVA, *P* = 0.08). Samples from non-progressor patients had the fewest somatic SV and CNV events, with between 2 and 16 SV events identified and less than 1% percent of the genome affected by copy number changes.

Telomere length was determined using qMotif (Fig. 1 e). There was no statistically significant difference in telomere length between the groups (ANOVA, *P* = 0.11). The majority of EAC samples (17/22) showed telomere shortening with a mean log2 telomere ratio of − 0.48 where zero represents a tumour telomere length that is equal to its matched normal. When examining all BE samples together, telomeres only exhibited a modest shortening (− 0.098). Three of five dysplastic samples had shorter telomeres, however the mean telomere length was close to zero: − 0.058. Most non-dysplastic samples also had shorter telomeres (6/7), with a mean of − 0.19. In non-progressor samples, one individual had shorter telomeres in samples from both Visit 1 and Visit 2, and the other individual had longer telomeres in both samples.

### Mutations in previously reported EAC genes

**Fig. 2:**
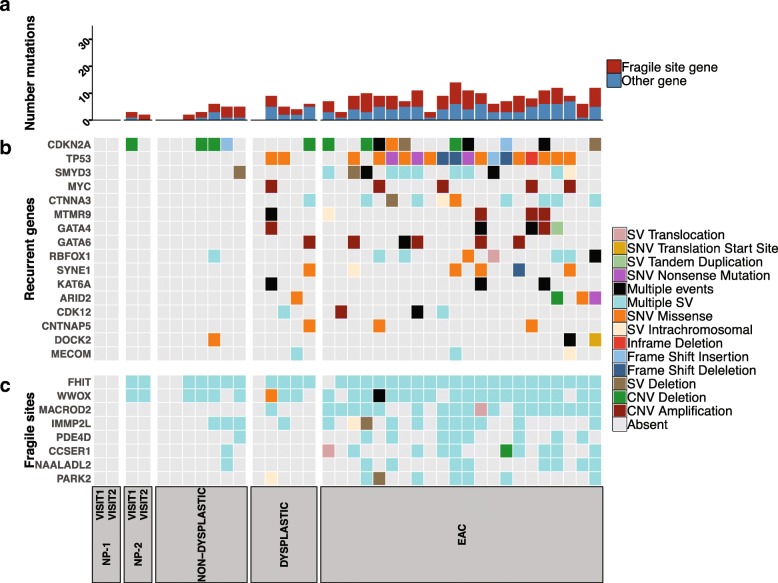
Mutations in previously reported EAC genes. The presence of SNVs, indels, copy number changes and structural variation in previously reported EAC genes [9, 11, 15, 35]. In the analysis, 74 genes were used. Of these, 24 genes were affected in at least one BE sample and are shown in the oncoplot. **a** Number of mutations in the 24 genes (**b**) Recurrent EAC genes that are not fragile sites (**c**) Mutations in fragile site genes. Sample order for the figure can be found in Additional file 2: Table S1c

We determined the EAC associated genes that were affected by somatic SNVs, indels, SVs and copy number deletions and amplifications. We concentrated on 73 genes that have been previously reported to be a driver or recurrently mutated in EAC (Additional file 3) [9, 11, 15, 35]. Twenty-four of these genes were mutated in at least one BE sample in the cohort (Fig. 2). Each EAC sample had mutations in 3–14 of these 24 genes. Fragile site genes (those listed as such by Secrier et al. [15]) were frequently affected by structural rearrangements: *FHIT* (21/22), *WWOX* (19/22), *MACROD2* (18/22) (Fig. 2 b). Other frequently mutated genes in the EAC samples that were also mutated in BE samples were *TP53* (17/22), *CDKN2A* (10/22), *SMYD3* (11/22) (Fig. 2 b).

Four of the 5 dysplastic BE samples also had mutations in 4–9 EAC reported genes, with one sample having no mutations in previously reported genes. Structural variant breakpoints in the fragile site gene *FHIT* were common, with mutations present in 4 samples. SVs were also present in 2 samples for *WWOX*. Mutations in other genes were more variable. *TP53* mutations were present in 2/5 dysplastic samples, whereas *CDKN2A* was only present in one sample. Notably, no loss of function *SMAD4* mutations were observed in the dysplastic samples.

Non-dysplastic samples exhibited mutations in only a few (range of 0–6 per sample) of the previously reported genes, with *CDKN2A* (3/7), *FHIT* (5/7) and *WWOX* (4/7) frequently mutated. No *TP53* or *SMAD4* mutations were observed in these samples. Non-progressor NP-1 had no mutations in genes previously reported to be mutated in EAC (Fig. 2). In the non-progressor NP-2 samples, *FHIT* and *WWOX* were mutated in samples taken at both Visit 1 and Visit 2 and *CDKN2A* in the visit 1 sample.

### Complex large-scale genomic events in BE samples

Complex genomic events such as chromothripsis and BFB are common in EAC [11, 15]. We therefore determined whether such events are present prior to the development of cancer. We examined the signatures of rearrangement mutational processes which have previously been identified in breast cancer, liver cancer as well as in EAC [15, 30, 32]. Analysis was performed using 32 subclasses previously described by Nik-Zainal et al. [30]. Rearrangements were classified based on whether rearrangements were clustered or not clustered, by rearrangement type (deletion, inversion, tandem duplication and deletion) and by size of the rearrangement. Due to low numbers of SV events, the non-progressor samples, and one dysplastic sample, which had only two SV events, were not used in the analysis.

**Fig. 3:**
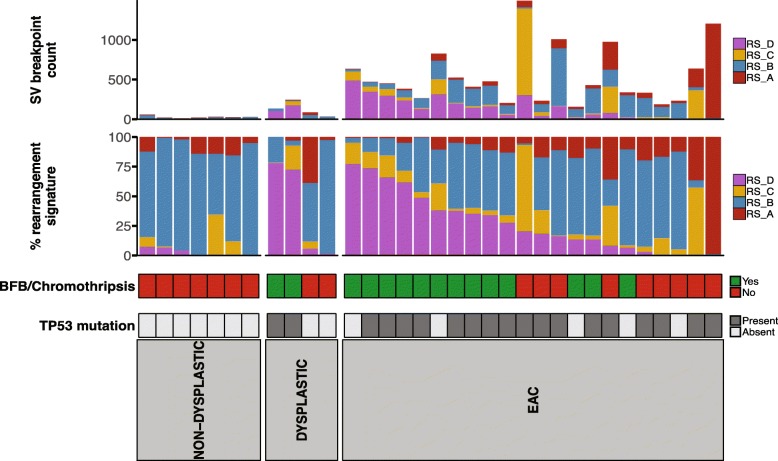
Contribution of rearrangement signatures to BE and EAC samples. The upper barchart shows the number of SV breakpoints and the contribution of the four rearrangement signatures to each sample. The lower barchart shows the contribution of each signature as a percentage. The presence of complex events that resemble BFB or chromothripsis and TP53 mutations is also shown. Non-progressor samples and one dysplastic sample are not shown due to having too few SV events to be used to identify signatures. Sample order for the figure can be found in Additional file 2: Table S1c

Four rearrangement signatures were identified within the EAC and BE cohort (Additional file 1: Figure S4). These signatures were compared against the six signatures identified by Nik-Zainal and co-workers (RS1–6) using cosine similarity. RS_A had a low correlation of cosine similarity of 0.65 with RS3 and 0.67 with RS5. RS_B was similar to RS2 (0.89) and RS_C was closest to RS3 (0.82). RS_D was not highly correlated, with the closest signature being RS4 (0.77) which represented clustered SVs. The rearrangement signature RS_D contributed to 0–78% of SV events within the samples (Fig. 3). Ten EAC samples had a contribution of the RS_D signature greater than 25% (Fig. 3). Two dysplastic samples had high contribution of the signature RS_D, in HGD-2 RS_D represented 73% of SVs and in HGD-3, 78%. These samples also had higher numbers of SV events in comparison with most other BE samples, with most SVs clustered in one or a few chromosomes. In contrast, other dysplastic BE and non-dysplastic BE had low or no evidence of RS_D, and lower numbers of SV events overall.

We then manually reviewed EAC and BE samples on a per-chromosome basis to determine if complex localised events were present. The EAC samples with a high contribution of RS_D frequently contained complex localised events that resembled chromothripsis and BFB (Fig. 3). Such complex events were also identified in our previous publication using these EAC samples [11].

**Fig. 4:**
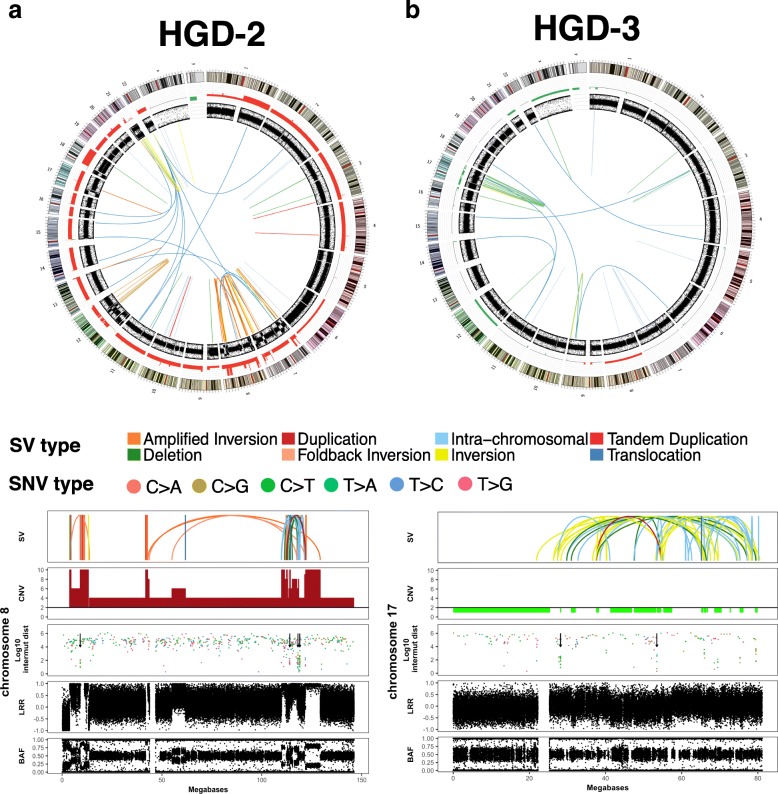
Complex events in dysplastic BE samples. **a** Complex events in sample HGD-2 (**b**) Complex events in HGD-3. For each sample**,** the upper panel shows a circos plot of structural variations (inner), B allele frequency (BAF), and copy number changes (green = loss, red = gain) according to chromosomal location (outer ring). The lower panels show events per chromosome (chromosome 8 in HGD-2 and chromosome 17 in HGD-3) with the top plot showing structural variations (colour coded according to the SV key shown); the second plot showing copy number changes (red = amplification, green = deletion). The third plot shows log10 inter-mutational distance between single nucleotide variants (colour coded according to the SNV key shown) with arrows indicating regions of kataegis. The fourth plot shows Log R ratio and the bottom plot shows BAF

In the two dysplastic BE samples with a high proportion of signature RS_D, we observed a number of features of localised complexity (Fig. 4). In patient HGD-2, chromosome 8 contained some characteristics of BFB including telomeric loss, a high concentration of inversions with 68 inversion breakpoints of the 104 breakpoints on the chromosome, and highly amplified regions (Fig. 4 a). Localised complex rearrangements with copy number amplifications, oscillating copy number and clustered SVs were also present on chromosome 7 (Additional file 1: Figure S5). The events on chromosome 8 are candidate driving events as they resulted in the amplification of known EAC genes, including *GATA4* and the oncogene *MYC.* In the dysplastic sample HGD-3, chromosome 17 had 91 SV breakpoints (62% of total breakpoints) and contained some of the characteristics of chromothripsis, including oscillating copy number and retention and loss of heterozygosity (Fig. 4 b). Both samples had mutations in *TP53* and evidence of either considerable or modest telomere shortening with a log2 telomere ratio of − 0.58 for HGD-2 and -0.1 for HGD-3.

Localised regions of base substitution hypermutation, known as kataegis, have previously been associated with clustered SV events [30], and are common in EAC [11]. We looked for the presence of kataegis in BE samples and observed that the two dysplastic samples with well-defined localised complex events also had kataegic loci (Fig. 4 a,b). HGD-2 had eight loci that were positive for kataegis, with 6 of these loci also located on chromosome 8 within the regions with some evidence for BFB. Of the remaining 2 loci, 1 region was located on chromosome 7, which also had local regions of complexity, and the other on chromosome 1. HGD-3 had 2 loci of kataegis, both of these on chromosome 17 in the vicinity of events that showed some evidence of chromothripsis.

We also observed some evidence for localised complexity in 2 other dysplastic samples, although there was insufficient evidence to define these regions as harbouring chromothripsis or BFB. Chromosome 4 of the dysplastic sample from HGD-4 had clustered SV breakpoints (14 breakpoints) and oscillating copy number (Additional file 1: Figure S6a). This sample had evidence for telomere shortening. For donor HGD-5, two chromosomes exhibited some localised complexity, with chromosome 5 showing regions of oscillating copy number, while chromosome 18 (Additional file 1: Figure S6b) contained 21 SV breakpoints and several highly amplified regions. This sample had no evidence of telomere shortening. This sample was the only dysplastic sample that was taken from a patient undergoing EAC tumour resection. It is therefore a region of BE adjacent to the EAC but was well separated from the tumour. Histology for the sample also confirmed the region of biopsy was high grade dysplasia and not EAC. In agreement with the low number of SV events and percentage of the genome affected by copy number changes, we did not observe any evidence of complex events in non-progressor samples or non-dysplastic samples (Additional file 1: Figure S7).

## Discussion

In this study, we characterised the whole-genomes of non-progressor, non-dysplastic and dysplastic BE samples and compared them with a previously described EAC cohort [11].

Overall, the mutation profile of BE samples agreed with previous studies of such samples. Although a higher burden of SNV/indels was observed in EAC compared with BE samples, no significant difference in the number of SNV/indel mutations was observed between non-dysplastic and dysplastic samples, either when examining all mutations or only coding mutations. The fact that the mutational burden is not statistically different between dysplastic and non-dysplastic samples may be due to the low number of samples in each cohort, however this observation is also in agreement with a previous study using whole-genome sequencing [10]. In contrast, Stachler and co-workers, using exome sequencing, found a significant difference in the number of SNV/indels between dysplastic and non-dysplastic samples [13]. Such differences observed between studies may be due to the difficulties of accurately identifying the dysplastic stage of BE by histopathology, as considerable inter-observer variability in the diagnosis of dysplasia in BE has been reported [36]. Alternately, as only strong evidence has been considered for the presence of a mutation, it is possible that dysplastic samples may have a more complex subclonal structure than non-dysplastic samples and therefore some mutations present in a lower number of cells could have been missed.

Mutations in previously reported EAC genes increased with progressing BE stages. The presence of *CDKN2A* and structural variations in fragile site genes *WWOX* and *FHIT* in non-progressor and other non-dysplastic samples agrees with previously published studies that suggest that these are very early events in BE [17, 23, 37]. The presence of *TP53* mutations in dysplastic samples but not non-dysplastic samples, and the absence of *SMAD4* mutations in BE samples agrees with the work of Weaver et al., who proposed that mutation of *TP53* marked the boundary from non-dysplastic to dysplastic and that *SMAD4* mutations are only present in invasive cancer [14]. However, *TP53* mutations have also been reported to be present in non-dysplastic BE biopsies taken in regions adjacent to EAC [10].

Previous studies have reported the copy number abnormalities of non-progressors and have demonstrated that they are stable over time [17]. The observation of structural variations in fragile site genes in both samples of the NP-2 non-progressor patient in this study agrees with Li et al. who saw similar chromosomal level events in non-progressors and that these events were stable across space and time [17]. The presence of *CDKN2A* loss in NP-2 also agrees with previous observations [17]. Less is known about the progression of SNV and indel mutations over time in non-progressor samples. In this study, we observed that non-progressor samples have between 273 and 2152 SNV/indel mutations on a whole-genome level, but few coding mutations, with none in previously reported EAC genes. In comparison, Weaver et al. observed a number of mutations in previously reported EAC genes, including *ARID1A* and *SMARCA4* when examining 66 non-dysplastic patients who had not progressed after a median follow-up time of 58 months [14]. We used two time points from two non-progressing patients to compare the overlap of mutations between the samples. When weak evidence for a variant was considered, the degree of overlap between samples was high, with 79 and 90% of SNV/indels common to both samples. For each non-progressor, each biopsy was taken from the same region, which may contribute to the generally high degree of overlap. Furthermore, the stability of SNV/indels present in these samples is consistent with the studies of copy number variation stability in non-progressors [19]. However, given the small number of samples and time-points examined in this study it is difficult to make definitive conclusions about the degree of overlap observed in these samples.

The presence of similar levels of Signature 17 in EAC and BE samples agrees with previous studies that have reported the presence of this signature in BE samples [10, 13]. It has been suggested that Signature 17 is due to oxidative damage [9], as a consequence of exposure to gastric and bile acids which occurs during chronic reflux. Therefore, mutations with the context for Signature 17 are likely to occur early in BE. Interestingly there was no evidence for Signature 17 in non-progressors, indicating that it is possible that these samples have been less exposed to the mutational processes, such as oxidative damage caused by reflux, or that the repair mechanisms that cope with this damage are intact. However, further analysis of a larger cohort of non-progressor samples is required to determine if this is a reproducible observation, particularly as in a targeted amplicon sequencing of 26 EAC genes, it was observed that 5/29 SNVs in non-progressors were T > G in a TT context, the context that is associated with Signature 17 [14].

We identified a rearrangement mutational signature that was present in many EAC samples that had complex genomic catastrophes including chromothripsis and BFB. Two dysplastic BE samples also had a high proportion of this signature. After manual review, the presence of complex rearrangements in these samples was confirmed, indicating the possible utility of rearrangement signatures to more easily identify samples with complex events. However, it should be noted that many of the BE samples only had a low number of SV events, and therefore rearrangement signature analysis in such samples could be limited.

In total, we found some evidence for complex events in four dysplastic BE samples, including two samples with some characteristics of BFB and chromothripsis, associated kataegic loci and some early signs of complex events in a further two samples. We have previously proposed that genomic catastrophes may be a possible mechanism whereby EAC arises rapidly in patients [11]. FISH experiments in medulloblastoma have also shown that chromothripsis events were not subclonal but were present in almost all tumour cells, suggesting that chromothripsis was an early event in tumorigenesis [38]. In support of this, Li and co-workers reported that 13 of 79 progressors (16.5%) had evidence of chromothripsis before the detection of EAC, as determined by SNP array [19] and whole-genome doublings and high genomic diversity have also been observed in patients within the 24 months prior to the detection of EA [17]. To our knowledge this study is the first to use whole-genome sequencing to identify evidence of BFB, chromothripsis and kataegis in BE samples.

Telomere shortening and *TP53* mutations are both proposed to be involved in the development of catastrophic events. *TP53* mutations have previously been reported to be associated with chromothripsis in medulloblastoma and acute myeloid leukaemia [38]. Chromothripsis has been proposed to arise via a number of mechanisms, with Maciejowski and co-workers reporting that both chromothripsis and kataegis can arise due to telomere crisis where telomere attrition leads to frequent telomere fusions, driving genomic instability [39]. The absence of functional p53 further contributes to telomere shortening and genomic instability as cells will progress into mitosis early with uncapped telomeres, leading to the tendency to generate end-to-end fusions [40]. Telomere shortening and *TP53* mutations are also both reported to be early events in BE [14, 18], and we have previously suggested these events may be a mechanism for driving genomic catastrophes in EAC [11]. The two dysplastic samples with *TP53* mutations had the strongest evidence for chromothripsis and BFB, and high levels of the RS_D rearrangement signature. These samples also had shorter telomeres. Therefore, dysplastic BE samples with *TP53* mutations and shortened telomeres may be more at risk of undergoing genomic catastrophes.

The presence of complex rearrangements in high-grade dysplastic samples in this study adds further support for these events to be considered drivers in the rapid progression from BE to EAC, despite the small number of samples examined. In addition, only high-grade dysplastic samples were described and no low-grade dysplastic samples were examined, so the existence of complex events in such samples is unknown. The ability to accurately define a specimen as high-grade dysplasia or adenocarcinoma is also problematic. Poor inter-observer reproducibility has been observed among pathologists when differentiating between high-grade dysplasia, intramucosal adenocarcinoma, and submucosal adenocarcinoma in a specimen [41] and it is possible that high-grade dysplastic samples with evidence of complexity may in fact already have progressed to adenocarcinoma. Therefore, the analysis of a larger cohort is required in order to confirm the observations made in this study.

## Conclusion

Overall, the genomic landscape of these BE samples agrees with previously reported data with respect to mutation burden, mutational signatures and telomere length. We have identified evidence for the presence of genomic catastrophes in high-grade dysplastic BE, and these samples also had shortened telomeres and *TP53* mutations. Therefore, these data provide further support for the hypothesis that the rapid progression from BE to EAC could be triggered by complex localised rearrangement events such as chromothripsis or BFB. However, due to the low number of samples used in this study, there is a need for a collaborative effort to source a larger cohort of BE samples with long term follow-up for whole genome sequencing in order to further characterise the genomic features of BE to EAC progression, in particular to confirm the presence of genomic catastrophes and the role that these events play in the development of invasive cancer.

## Additional files

Additional file 1: **Figure S1.** Non-progressor sample collection time line**. Figure S2.** Overlap of SNV/Indels in non-progressor samples**. Figure S3.** Mutational signatures in EAC and BE samples**. Figure S4.** Structural rearrangement signatures in EAC and BE samples**. Figure S5.** A complex event on chromosome 7 in the dysplastic sample HGD-2**. Figure S6.** Other complex events in dysplastic BE samples. **Figure S7.** Distribution of SV and copy number events in non-dysplastic BE samples. **Figure S8.** Representative images of Barrett’s oesophagus. (PDF 2260 kb)

Additional file 2: **Table S1a**. Clinical information for 22 EAC samples. **Table S1b**. Clinical information for 16 BE samples. **Table S1c.** WGS coverage metrics and order of samples for Figs. 1, 2 and 3. (XLSX 17 kb)

Additional file 3: List of genes previously implicated in EAC. (PDF 17 kb)

## Abbreviations

BAF: B allele frequency
BE: Barrett’s oesophagus
BFB: Breakage-fusion-bridge
CNV: Copy number variants
EAC: Oesophageal adenocarcinoma
HGD: High-grade dysplasia
LGD: Low-grade dysplasia
LRR: Log R ratio
NMF: Non-negative matrix factorization
SNV: Single nucleotide variant
SV: Structural variant
