# Supplementary material for: Complex structural rearrangements are present in high-grade dysplastic Barrett’s oesophagus samples
Source: BMC Med Genomics. 2019 Feb 4;12:31. doi: 10.1186/s12920-019-0476-9 (PMC6360790; doi:10.1186/s12920-019-0476-9)
Supplement: Supplementary file 1 — Figure S1. Non-progressor sample collection time line. Figure S2. Overlap of SNV/Indels in non-progressor samples. Figure S3. Mutational signatures in EAC and BE samples. Figure S4. Structural rearrangement signatures in EAC and BE samples. Figure S5. A complex event on chromosome 7 in the dysplastic sample HGD-2. Figure S6. Other complex events in dysplastic BE samples. Figure S7. Distribution of SV and copy number events in non-dysplastic BE samples. Figure S8. Representative images of Barrett’s oesophagus. (PDF 2260 kb) [file 12920_2019_476_MOESM1_ESM.pdf]

Supplementary Figures

Figure S1

Non-progressor sample collection time line

Figure S2

Overlap of SNV/Indels in non-progressor samples

Figure S3

Mutational signatures in EAC and BE samples

Figure S4

Structural rearrangement signatures in EAC and BE samples

Figure S5

A complex event on chromosome 7 in the dysplastic sample HGD-2

Figure S6

Other complex events in dysplastic BE samples

Figure S7

Distribution of SV and copy number events in non-dysplastic BE samples.

Figure S8

Representative images of Barrett’s oesophagus

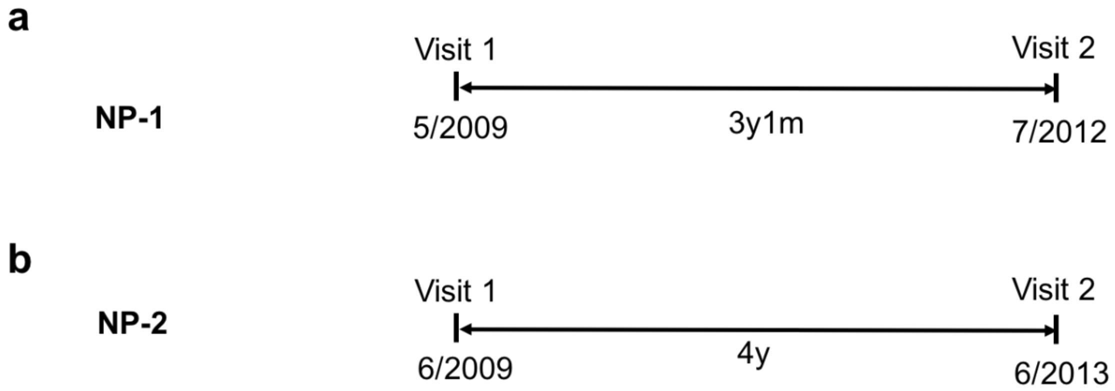

Figure S1: Non-progressor sample collection time line. a. Non-progressor patient 1**,** NP-1 Visit 1 (NP-1-1) sample was collected in May 2009 and the Visit 2 sample (NP-1-2) is from Jul 2012, 3 years and one month later. The patient was alive in 2016, but no other biopy is available. **b**. Non-progressor patient 2, NP-2 Visit 1 (NP-2-1) sample was collected in June 2009 and the Visit 2 (NP-2-2) sample was collected 4 years later in June 2013. Patient was followed for a total of 8 years, with a biopsy in 2017 (four years after the visit 2 sample that was sequenced), showing no evidence of dysplasia.

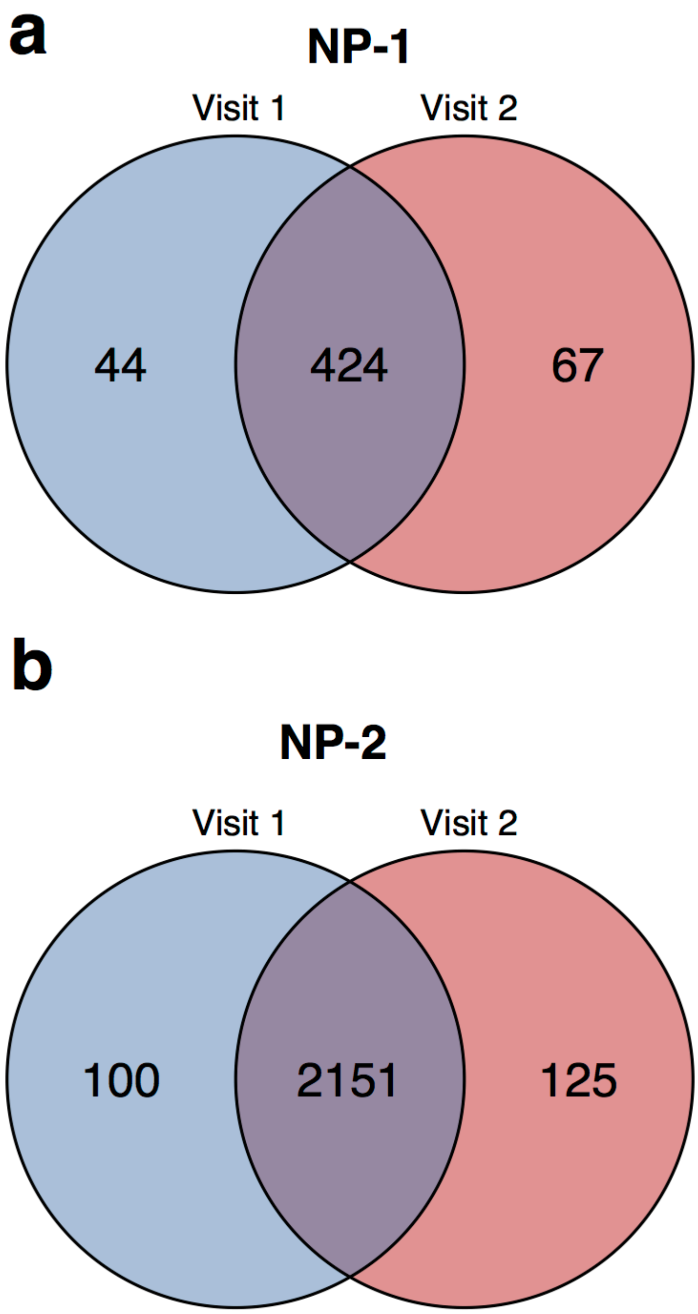

Figure S2: Overlap of SNV/Indels in non-progressor samples. Venn diagrams showing overlap in SNV/indels detected across the whole genome between Visit 1 (left in blue) and Visit 2 samples (right in red)**. a** Non-progressor patient 1, NP-1. **b** Non-progressor patient 2, NP-2.

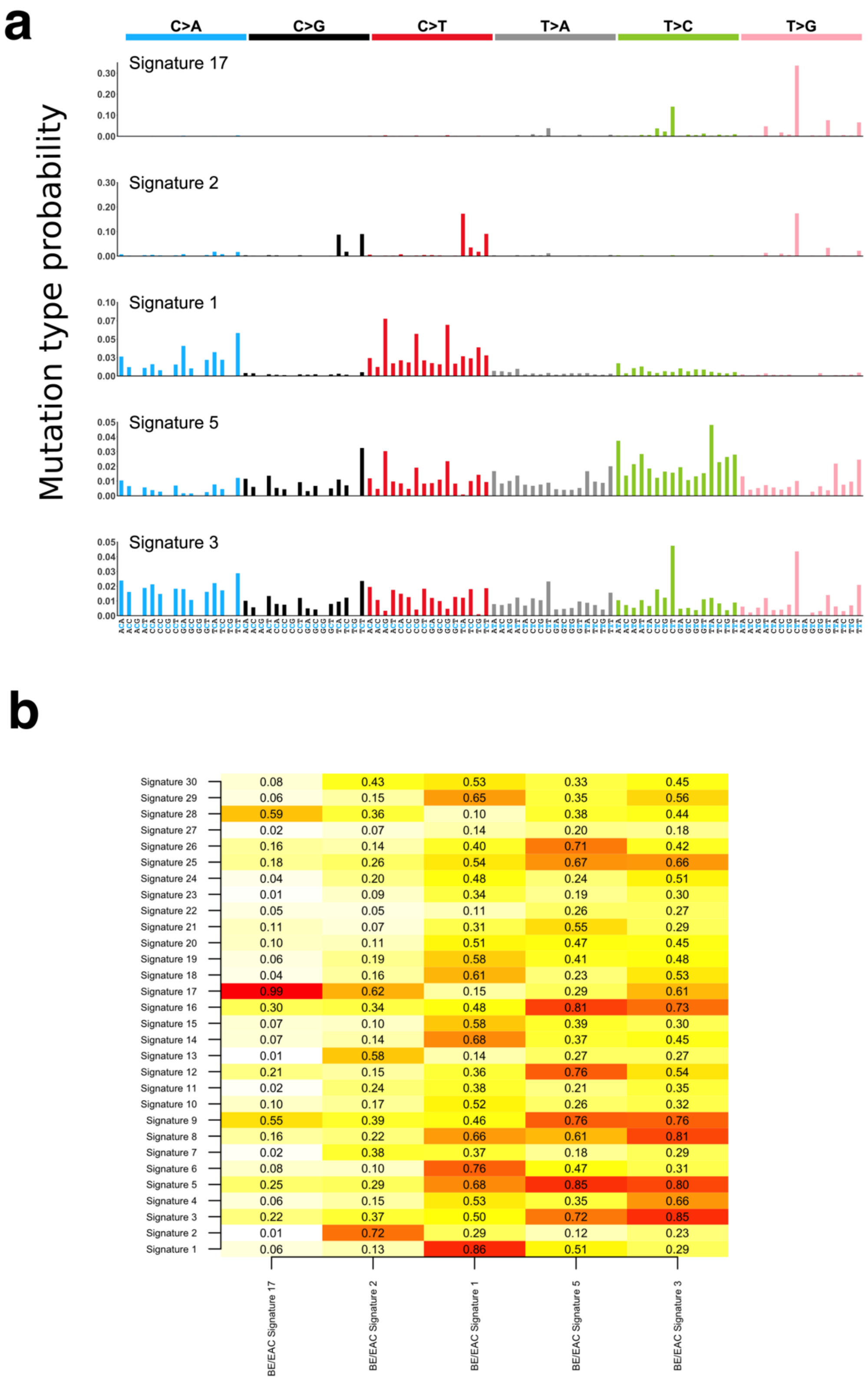

Figure S3. Mutational signatures in EAC and BE samples. a Five signatures were identified. Each signature is represented by the mutational type probability of each substitution in a trinucleotide context (96 contexts). **b** Cosine similarity with COSMIC signatures.

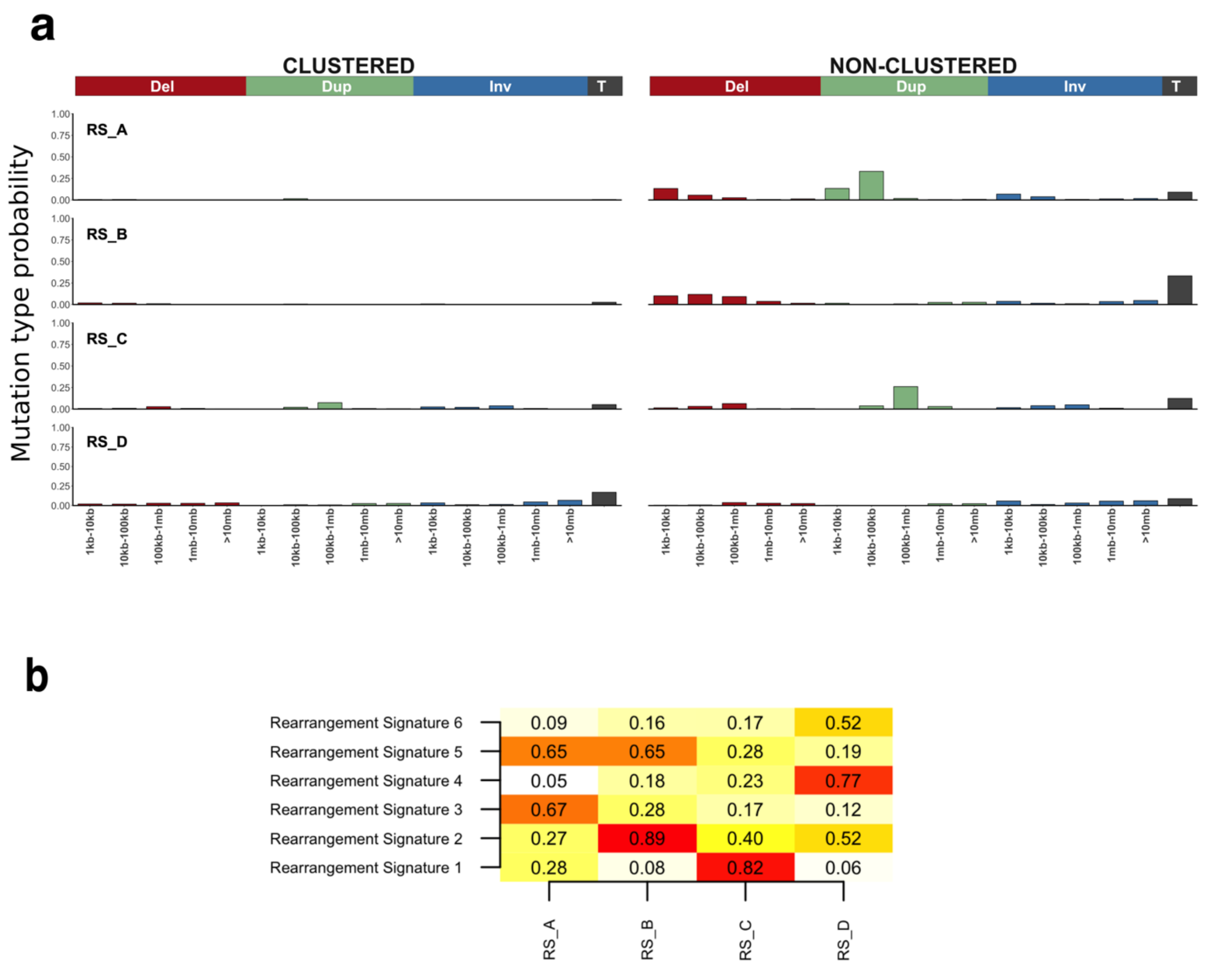

Figure S4. Structural rearrangement signatures in EAC and BE samples. a Four signatures were identified by NMF (RS_A to RS_D). Rearrangements were classified into 32 categories by type (Del=deletion, Dup=duplication, Inv=inversion, T=translocation), size which is detailed in the x-axis and whether breakpoints were clustered (left) or non-clustered (right). The y-axis represents the probability for each rearrangement category. **b** Cosine similarity with Nik-Zainal et al signatures.

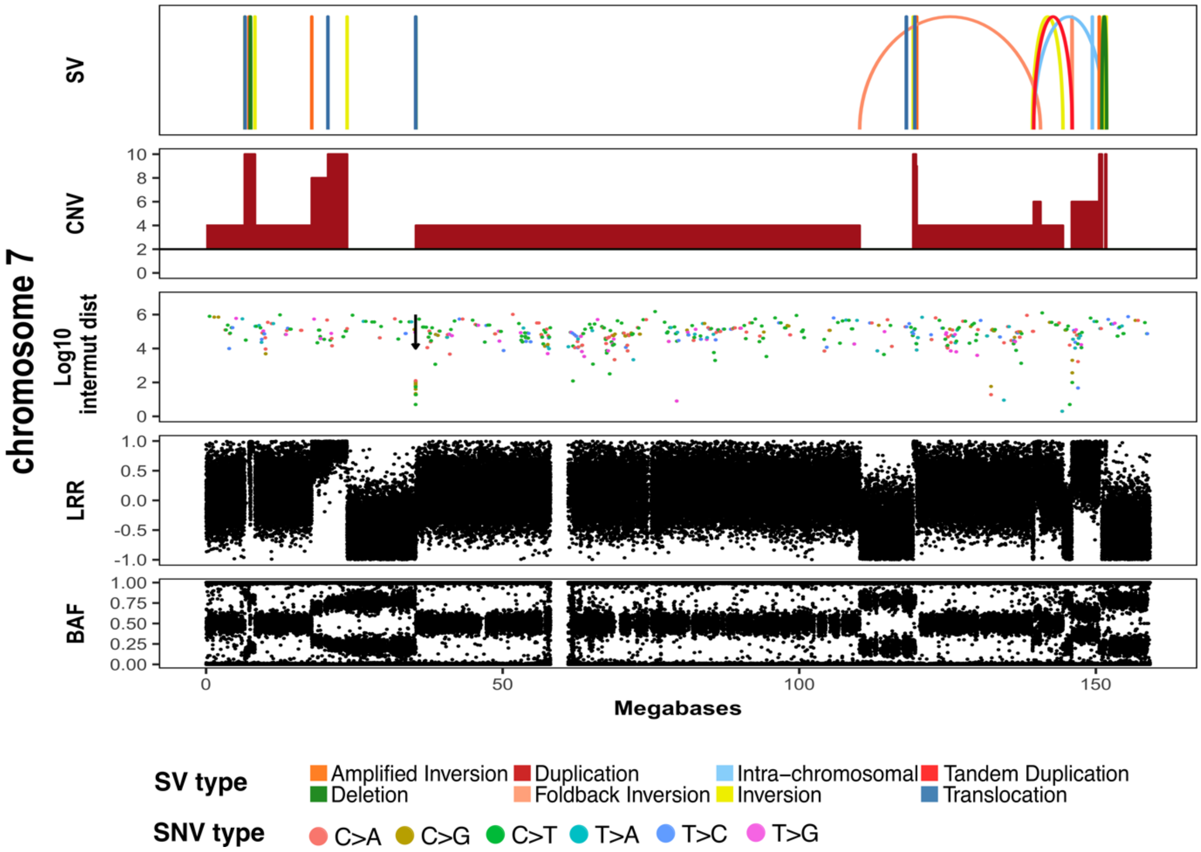

Figure S5. **A complex event on chromosome 7 in the dysplastic sample HGD-2.** The top plot shows structural variations (SV); the second plot shows copy number variants (CNV, red=amplified). The third plot shows log10 inter-mutational distance between single nucleotide variants (SNVs). Arrow points to a region of kataegis. The fourth plot shows Log R ratio (LRR) and the bottom plot shows B allele frequency (BAF). Chromosome 7 has a region of localised complexity with clustered SV breakpoints and high copy number (≥10) amplifications.

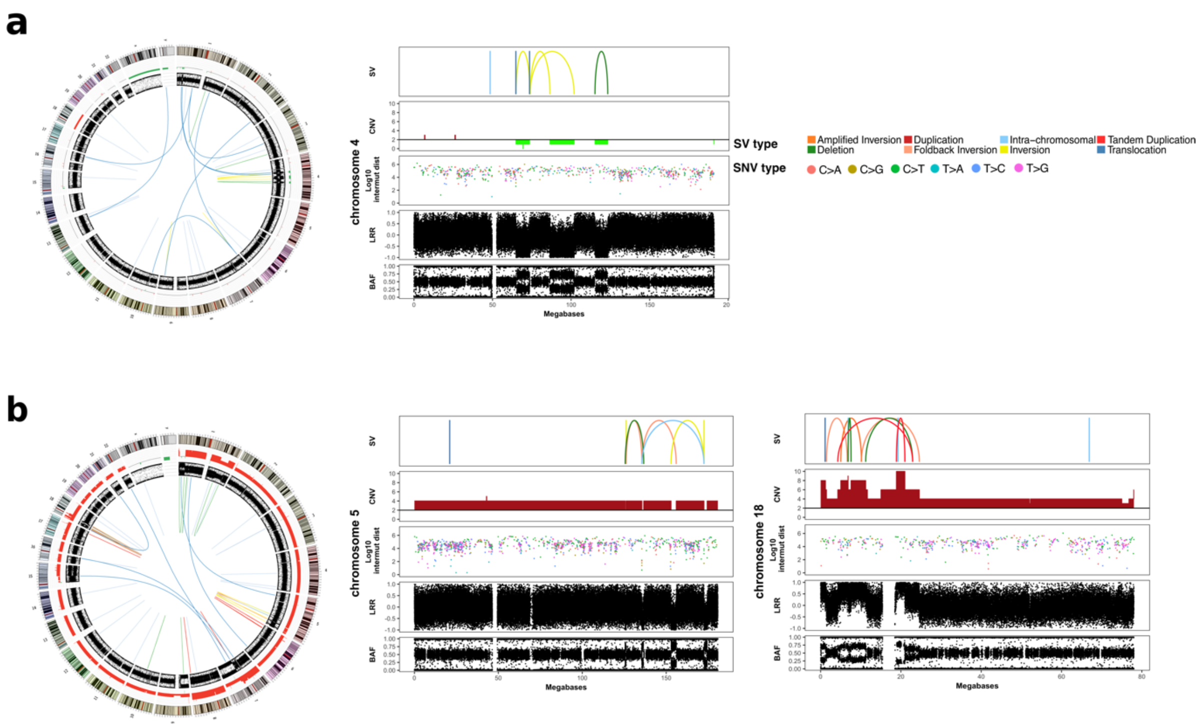

Figure S6. Other complex events in dysplastic BE samples. a Circos plot of HGD-4 showing structural variant (SV) events (inner, colour coded according to the chart on the right) and copy number and B allele frequency (BAF, outer). The panel on the right shows chromosome 4 of HGD-4 with regions of clustered SVs, oscillating copy number, and loss (green) and retention of heterozygosity. **b** Circos plot of HGD-5 and individual chromosome plots of chromosome 5 and 18. Chromosome 5 shows a region of clustered SVs, oscillating copy number, and loss and retention of heterozygosity. Chromosome 18 shows a region of clustered SVs and copy number amplifications (red). CNV=copy number variant, LRR=Log R ratio, SNV=single nucleotide variant.

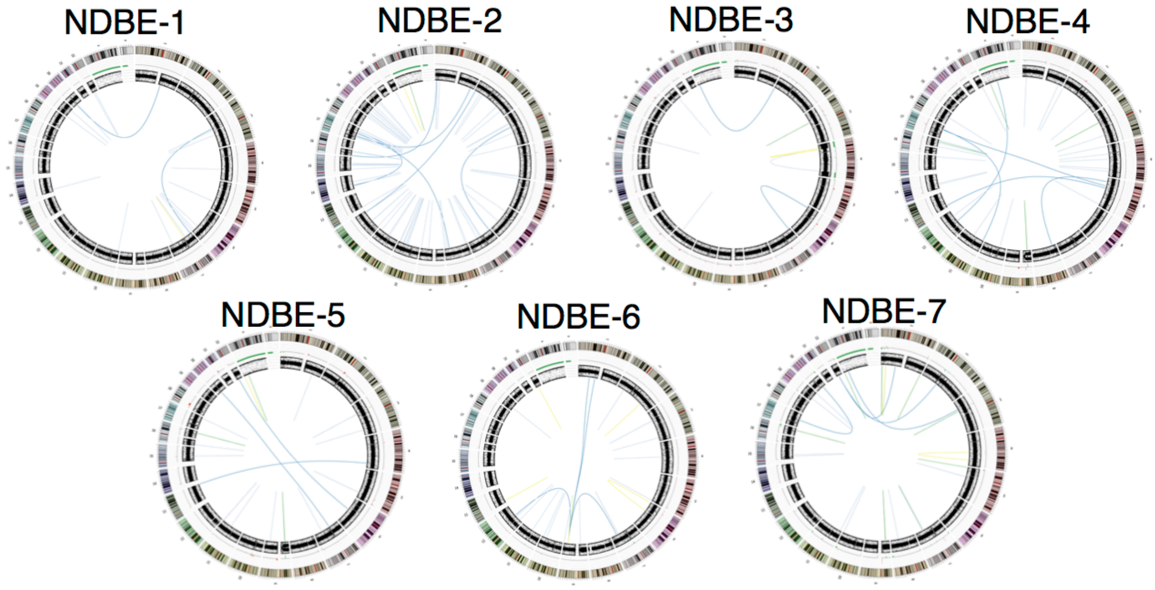

Figure S7: **Distribution of SV and copy number events in non-dysplastic BE samples.** The circos plot for each sample shows structural variants in the centre, B allele frequency (BAF) and copy number (red/green), arranged according to chromosomal location (outer ring).

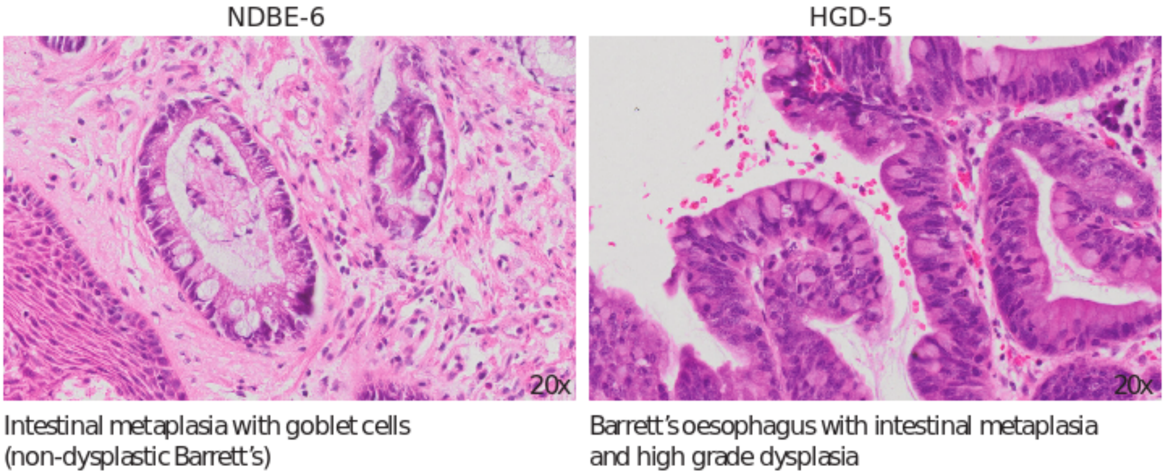

Figure S8: Representative images of Barrett's oesophagus. Representative image of non-dysplastic Barrett's oesophagus (NDBE-6, left) and representative image of dysplastic Barrett's oesophagus (HGD-5, right)
