# Supplementary material for: Complex structural rearrangements are present in high-grade dysplastic Barrett’s oesophagus samples
Source: BMC Med Genomics. 2019 Feb 4;12:31. doi: 10.1186/s12920-019-0476-9 (PMC6360790; doi:10.1186/s12920-019-0476-9)
Supplement: Supplementary file 3 — List of genes previously implicated in EAC. (PDF 17 kb) [file 12920_2019_476_MOESM3_ESM.pdf]

Previously reported driver or recurrently mutated genes in EAC

ACTL7B
ABCB1
ABGL4
AJAP1
AKAP6
ARID1A
ARID2
C6orf118
CCDC102B
CCNE1
CCND1
CCSER1
CDH18
CDK12
CDK14
CDKN2A
CNTNAP5
CTNNA2
CTNNA3
CYP7B1
DOCK2
EGFR
ELMO1
ERBB2
ERBB4
EYS
F5
FHIT
FRS2
GATA4
GATA6
HECW1
IMMP2L
IGFR1
JUP
KAT6A
KCNQ3
KCNU1
KIP26B
KRAS
MTMR9
MECOM
MYC
MYO18B
MACROD2

MDM2
NAALADL2
NUAK1
PARK2
PARKD3B
PIK3CA
PDE4D
PRKG1
PBRM1
RB1
RBFOX1
RUNX1
SCN10A
SLC39A12
SMAD4
SMARCA4
SMYD3
SAMD5
SASH1
SOX5
SPG20
SYNE1
THADA
TLL1
TLR4
TP53
VEGFA
WWOX
